# A sociocultural perspective of mental health stigma in Malta

**DOI:** 10.3389/fpsyt.2023.1229920

**Published:** 2023-08-03

**Authors:** Josianne Scerri, Alexei Sammut, Janice Agius

**Affiliations:** ^1^Department of Mental Health, Faculty of Health Sciences, University of Malta, Tal-Qroqq, Malta; ^2^Faculty of Health, Science, Social Care and Education, Kingston University, London, United Kingdom

**Keywords:** mental health stigma, mental health in Malta, sociocultural perspective, service evolution, stigma reduction, stigma attitudes

## Introduction

Epidemiological research depicting the overall prevalence of mental health disorders in Malta is sparse. With a population of over 535,000 inhabitants, it is estimated that around 120,000 individuals have a mental disorder (1). The reported local percentage prevalence stands at 6.6% for depression; 7.8% for anxiety, and for schizophrenia, at 0.026% for the general population and 0.4% for asylum seekers (2, 3). Approximately 25.2% of individuals under the age of 14 are at risk of developing a mental disorder, which is higher than that estimated in Europe (3, 4).

Stigma, or the negative judgement toward individuals with a mental illness (5), is a prevailing concern that has been on local and international agendas for years. Research worldwide depicts that stigma affects various dimensions, including treatment adherence, family dynamics, employment, social inclusion, and the occurrence of other mental health illnesses (5–8). Individuals having mental illnesses in Malta also experience stigma (9), and an exploration of this phenomenon requires immersion in a Mediterranean cultural context, due to its influence on Maltese society. Such cultural beliefs are of significance as they influence how disorders are understood, described, and managed, how help is sought and how treatment is received (10). Malta, being at the center of the Mediterranean Sea, and having been under the governance of Arabic, Central and Southern European territories, has been inspired to develop into what it is today, with Mediterranean values remaining prominent. Familial support, honor, religion, shame, and strong family values are factors shaping overall wellbeing in a Mediterranean culture, possibly affecting how Maltese society views and behaves when confronted with mental illness (7).

Despite efforts to shift institutional care to community care, discussing mental health disorders is still not a customary practice and many refrain from admitting to any pertinent mental health challenges. Conversely, discussing mental health wellbeing in general is becoming more acceptable, possibly due to the ever-increasing mental wellbeing awareness campaigns, focusing on the importance of creating a healthy work-life balance. The association between Maltese cultural and societal norms and public attitudes toward mental health poses concern, particularly when trying to curb stigmatizing behaviors. This article aims at raising awareness on the impact of this relationship and offers views on the effectiveness of current stigma reduction movements and initiatives.

## Stigma on an individual level

Locally, stigma has been perceived as affecting the wellbeing of people with mental illnesses, often leading to discrimination, marginalization, and a general label that they are harmful to society (11). One area which stigma affects deeply is the self, as individuals with mental illnesses come to believe stereotypes and stigmatizing attitudes, such as being weak, unworthy, or incapable of succeeding in life. The internalization of such beliefs is referred to as self-stigma (11–13), and often results in feelings of hopelessness, low self-esteem and low self-efficacy. This lack of self-belief has long been recognized as having a negative impact on recovery processes, independent living, empowerment, and the development of social interactions (14), creating a vicious cycle that further validates public stigma.

It is crucial to interpret the impact of self-stigma alongside sociocultural and sociopolitical contexts of a specific population (13). Different populations develop different forms of stereotypes, however growing up with conceptualized ideas of mental illness increases the risk of self-stigma (14). For instance, the importance of upholding family honor by avoiding familial shame, remains prominent in a Maltese sociocultural context and hinders many from seeking the required help (15). Experiences of mental illness are often denied and not spoken about, especially within communities harboring strong stereotypes, for fear of familial exclusion or deterioration in relationships. In fact, locals take an average of 6.25 years before seeking help, with fear of isolation, embarrassment, and shame being significant contributing factors (16, 17).

Self-stigma is also reported to negatively impact interactions with professionals and relatives alike (18). Health professionals may perceive self-stigma in people with mental disorders, as triggering a sense of powerlessness and avoidance of situations where they encounter discrimination (13). They may also view self-stigma as a choice rather than customary practice. This phenomenon, better known as iatrogenic stigma, can exacerbate structural stigma, in that forms of discrimination and downgrading are often transferred onto the workplace. With Malta being a small island, this problem is further intensified, as many inhabitants are related or know each other personally. Galea (19) details how structural stigma manifests in Malta, highlighting issues of maltreatment, lower wages, lower advancement opportunities and an inability to find jobs that they are qualified in, attesting the cycle of self-stigma. Such issues often lead to poverty, and generally relatives feel duty-bound to support their relative with mental health challenges financially, causing further emotional and physical strain on relatives, who may need to work multiple jobs to make ends meet.

## Stigma on a communal level

Overall Maltese society still endorses the importance of maintaining close family networks, from which support, be it emotional, moral or financial can be received. In neighborhoods sustaining the sense of traditional community, support may also be sought from neighbors and other members of that community, who are often considered as extended family. This support can easily be shattered if people with mental illnesses are ostracized for fear of bringing shame to the family or the community. Such disregard can further deteriorate mental wellbeing, increase dependence on social welfare and increase self-stigma (7).

Nonetheless, one still finds families who choose to walk the recovery journey with their relatives. Fenech and Scerri (20) describe the emotional turmoil of caring for a relative with a mental illness, as well as the potential negative impact on the caregivers' well-being due to lifestyle changes and added responsibilities. This study highlights the family's role in caring, providing support and assisting with coping, a value deeply endorsed by Mediterranean cultures and still present in Malta today. As participants hereby stated, it is often either the parents or a person (such as a sibling) perceived as having the least commitments who take on the caring role, and a shift in responsibilities onto other relatives tends to be done with hesitancy. The impact of stigma upon social inclusion, acceptance and employment was also emphasized, noting how full integration into society presents a significant challenge. This study further depicts the financial strain imposed upon caregivers as a result of their relative's difficulty in finding employment.

Derogatory comments toward people with mental illness remain common, affecting not just self-perception, but also help-seeking attitudes. The Maltese term “mignun” has been repeatedly used to refer to someone experiencing a mental illness (21). It is not uncommon for locals to feel unsafe when seeking professional help, out of fear that even professionals might show stigmatizing and condemnatory approaches. Locally, iatrogenic stigma, manifests in various forms, for instance the diagnostic labeling of individuals with mental illness, and the notion that individuals with certain conditions, such as substance misuse, are inclined toward aggression or service manipulation. In the study by Galea (19), participants who experienced iatrogenic stigma recounted how professionals delimit people with mental illness, believing that they cannot do much on their own, and exhibit paternalistic and patronizing behaviors. Consequently, many locals interpret that symptoms of a psychological nature have a primary physical cause, compelling them to first seek medical help, unconsciously or deliberately avoiding psychological support. Furthermore, this may be attributed to the locals' close link and faith in primary healthcare, particularly their family doctor, who is often their sole point of reference for anything medically related.

Cachia (21) also emphasized stigma endured by children and adolescents. School-based professional services, aimed at promoting help-seeking attitudes and increasing mental health literacy from a young age are on the rise. Nevertheless, such services can have a rebound effect and discourage use, out of fear of being discovered by peers and possibly social group exclusion (19). Discordance between children and their parents has also been considered as barriers to help-seeking (22). Further misconceptions and negative prejudice toward mental health in general may reflect the population's dearth of mental health literacy.

## The evolution of services

Mental healthcare in Malta remains somewhat hospital-based and medically driven, with comparatively less attention given to alternative/complimentary therapies. Geographically, Malta is an archipelago of small islands, and there is one psychiatric hospital that caters for the mental health needs of the population. Having been built in the 19^th^ century, this building is still referred to by a stigmatizing term, that continues to fuel the everyday stigma experienced by individuals requiring hospitalization (19). The lack of sufficient investment in maintaining and improving human and infrastructural resources has created challenges in offering quality mental healthcare (3).

Over the years, however, there has been much investment allocated to developing community-based mental health services such as mental health clinics and outreach teams, that offer services closer to people and that help reduce stigma (23). These clinics are distributed across the island and hence vary in relation to catchment area and the number of persons being cared for. They also provide better access to mental health services, as requests for mental healthcare are directly referred to them, without having to unnecessarily go through the inpatient pathway. Despite this, the increasing demand for human resources in community-based services poses a challenge to provide adequate support, leading to unnecessary prolonged hospital stays. In fact, the average length of stay in Malta is one of the highest in Europe and has increased over the past few years (3).

In 2012, a New Mental Health Act came into effect. This law provided people having a mental illness with civil, political, economic, social, religious, educational, and cultural rights, which were previously unheard of, such as the right to actively participate in care and the right to select a responsible carer of one's own choice. This law significantly reduced the length of stay for involuntary care, such that an involuntary admission for treatment order was reduced from one year to 10 weeks (24).

The Office of the Commissioner for Mental Health (OCMH) was also subsequently established to safeguard clients' rights and ensure that the commitment toward advancement of mental health services remains (25). In 2019, this Office pushed for a 10-year Mental Health Strategy. Regardless of governmental commitment toward this strategy, investment remains primarily focused on improving medical care. It is difficult to determine whether this is due to stigma, or the impact of the COVID-19 pandemic, with a focus on prevention, containment, and management. Nonetheless, the percentage of Members of Parliament participating in annual debates organized by the OCMH, remains at an average low of 10% or less (17, 25, 26). Such statistics may provide a twofold indication; primarily of how governmental bodies perceive mental wellbeing; and the persistent stigma surrounding mental health.

The total number of trained mental health professionals remains amongst the lowest in Europe. The Maltese Association of Psychiatrists highlighted the low number of trained professionals and the large discrepancy between current and recommended practicing numbers (25, 27). The low number of prospective students also reflects the fear of stigmatizing attitudes by peers and colleagues. This inclination has recently started shifting, with more nurses working in specializations other than mental health, recognizing the importance of mental health literacy and engaging in specialized training.

## Mental health promotion

The relationship between low mental health literacy and stigma has long been investigated (5, 6). Recent research examining the likelihood of individuals seeking support following social media promotion has instigated the commencement of several movements and campaigns, aiming at increasing mental health literacy (28).

Locally, World Mental Health Day is now being celebrated yearly, with the OCMH organizing events to promote mental wellbeing. Throughout the year, the OCMH frequently discusses current affairs having a direct or an underlying mental health theme at different media houses. Similarly, several professionals participate in debates on local media, discussing stigma, services offered, mental illnesses, and holistic factors that contribute to mental wellbeing. In 2018, the #StopStigma national campaign endeavored to normalize and equalize mental health care. This campaign saw the OCMH, academics working within the University's mental health department and students undertaking the mental health nursing course create a series of informative posters that were distributed nationally.

Promotion is also being done within the education system. Children are being informed about the importance of maintaining mental wellbeing, accepting peers from diverse cultural or socioeconomic status, and sheering away from behaviors that can precipitate addiction. There is also increased awareness about the importance of early identification of autism, ADHD and other mental health disorders. Nurture classes and support zones have been developed in primary and secondary schools respectively, whereas students at tertiary-level education can access mental health services on campus (22).

One local non-governmental organization commenced training on “Mental health first aid,” with the aim of increasing knowledge and providing participants with skills to support individuals experiencing mental health issues. Following an initial target of students and educators, it is nowadays tailormade to various sectors including businesses, healthcare, and disciplined corps.

Public self-disclosures, previously considered implausible and taboo, have recently increased in settings such as media houses, self-help groups, seminars, and schools. Most self-help groups, as those for substance misuse, anxiety, depression and psychosis, are delivered by individuals on the path to mental health recovery. Others have developed their own nongovernmental organization, such as that for bipolar disorder, named “Be Positive, Bipolar Self-Help Malta.” Public disclosure, however, has its challenges. Instances whereby individuals were dismissed from work or long-term unemployment have been reported, making it difficult for them to live a flourishing and fulfilling life. Fears for public disclosure are recently being challenged by the contribution of foreigners experiencing mental health problems, whose own culture may possibly view this as an opportunity to influence a wider audience.

Co-production and service-user movements are still in their infancy in Malta, but initiatives are being made. The voices of experts by experience in relation to the formulation and delivery of some courses and care provision are now being sought. Nonetheless, further efforts are required for their inclusion in the formulation, implementation, and evaluation of policies. Two factors possibly affect co-production, iatrogenic stigma, and structural stigma (29, 30). People with mental health challenges still need to face the daily reality of lack of adjustment and understanding from employers, hindering full and active participation within society, including co-production movements. Despite this, the Alliance for Mental Health (A4MH), which consists of various mental health stakeholders, has discussed, and documented at length the need for service improvement, delivery, and access. Well-intentioned improvements, it states, can only be generated by prioritizing the involvement of clients and caregivers (31).

## Discussion

Mental health stigma remains a debilitating issue worldwide, the extent and intensity of which is affected by cultural and societal factors. Within the Maltese context there is still a dearth of local research highlighting the multifactorial considerations and complications of stigma. Although local promotion campaigns have multiplied, their impact is yet to be measured. Concern over society's unaddressed mental health needs due to stigma, and the consequent effect on the population's general health have also been highlighted (27).

Mental health stigma can be reduced, but it must be targeted in a systematic manner (6). One cannot change society's views on mental health without tackling iatrogenic stigma first. Incentives to increase professionals' interest in furthering related education need to be devised and implemented. Recruitment within mental health has become a global phenomenon, and enrollment efforts are being dampened by stigmatizing attitudes. Despite this, recruitment efforts have intensified and evolved in an attempt to reach a wider population. Adequate and quality person-centered care cannot be delivered if professionals keep demonstrating stigmatizing attitudes and authoritarian approaches to care.

Continuous education and public campaigns normalizing mental wellbeing need to become standard, as does professional support, particularly in school-based, university and workplace settings (30). Education needs to be further strengthened through the practice of informal interaction between service-users and general public (6).

Despite a shift in favor of normalization, mental health stigma remains prominent and tangible within the Maltese context. It is hoped that by targeting mental health stigma, the fear, shame, and negative beliefs surrounding mental health decrease.

## Author contributions

JS, AS, and JA contributed to concept, design of work, and wrote sections of the manuscript. All authors contributed to manuscript revision, read, and approved the submitted version.

## References

[1] World Health Organization. World Mental Health Day 2020: Malta Launches Campaign – “Move for Mental Health: Let's Invest!”. (2020). Available online at: https://www.who.int/europe/news/item/16-10-2020-world-mental-health-day-2020-malta-launches-campaign-move-for-mental-health-let-s-invest- (accessed May 1, 2023).

[2] Grech A. Epidemiology of serious mental illness in Malta – consequences for developing a new psychiatric hospital and community psychiatry. Psychiatr Danub. (2013) 28:108–10. 27663818

[3] Office of the Deputy Prime Minister. Building resilience, transforming services – A mental health strategy for Malta 2020-2030. (2018). Available online at: https://meae.gov.mt/en/Public_Consultations/MEH-HEALTH/Documents/Mental%20Health%20Strategy.pdf (accessed April 25, 2023).

[4] Sacco R Camilleri N Newbury-Birsch D. National study on mental health and emotional wellbeing among young people in Malta: Phase 1. Eur Psychiatry. (2022) 65:S596–7. 10.1192/j.eurpsy.2022.1527

[5] Simmons L Jones T Bradley E. Reducing mental health stigma: The relationship between knowledge and attitide change. Eur J Ment Health. (2017) 12:1–5. 10.5708/EJMH.12.2017.1.231030696

[6] Klaric M Lovric S. Methods to fight mental illness stigma. Psychiatr Danub. (2017) 29:910–7. 29283989

[7] Satariano B Curtis SE. The experience of social determinants of health within a Southern European Maltese culture. Health Place. (2018) 51:11. 10.1016/j.healthplace.2018.02.011 29549753

[8] World Health Organization. WHO *European Framework for Action in Mental Health 2021-2025: Draft for the Seventy-first Regional Committee for Europe*. (2021). Available online at: https://www.who.int/europe/publications/i/item/9789289057813 (accessed February 26, 2023).

[9] Agius M Falzon Aquilina F Pace C Grech A. Stigma in Malta: a Mediterranean perspective. Psychiatr Danub. (2016) 28:75–8. 27663810

[10] Molloy L Beckett P Chidarikire S Merrick TT Guha M Patton D. Culture, the stigma of mental illness, and young people. J Psychosoc Nurs. (2020) 58:15–8. 10.3928/02793695-20201013-03 33119117

[11] Oexle N Müller M Kawohl W Xu Z Viering S Wyss C. Self-stigma as a barrier to recovery: a longitudinal study. Eur ArchPsychiatry Clin Neurosci. (2018) 268:209–12. 10.1007/s00406-017-0773-2 28188369

[12] Corrigan PW Andrea B. Bink AB, Schmidt A, Jones N, Ruesch N. What is the impact of self-stigma? Loss of self-respect and the “why try” effect. J Ment Health. (2016) 25:10–5. 10.3109/09638237.2015.1021902 26193430

[13] Armstrong V Brandon T. Mental distress and “self-stigma” in the context of support provision: exploring attributions of self-stigma as sanism. Ment Health Soc Incl. (2020) 24:41–8. 10.1108/MHSI-09-2019-0028

[14] Corrigan PW Watson AC Barr L. The self-stigma of mental illness: Implications for self-esteem and self-efficacy. J Soc Clin Psychol. (2006) 25:875–84. 10.1521/jscp.2006.25.8.875

[15] Buttigieg A. Factors Influencing Help-Seeking for Mental Health Problems in Adolescents. [master's thesis]. Malta: University of Malta (2015).

[16] Grech A. Mental Health Literacy, Stigma and Attitudes Towards Seeking Help in Malta. [master's thesis]. Malta: University of Malta (2019).

[17] Cachia J. “*Empowering Stakeholders: Building Bridges and Crossing Them Together”: Annual Report 2018*. (2019). Available online at: https://healthservices.gov.mt/en/CommMentalHealth/Documents/Annual%20Report%202018.pdf (accessed March 3, 2023).

[18] Dobransky K. Reassessing mental illness stigma in mental health care: Competing stigmas and risk containment. Soc Sci Med. (2020) 249:2861. 10.1016/j.socscimed.2020.112861 32087486

[19] Galea R. The Lived Experiences of People With Mental Illness and the Impact on Their Families. [Master's thesis]. Malta: University of Malta (2017).

[20] Fenech M Scerri J. The impact of providing care to relatives with a severe mental illness: The caregivers' experience. Malta J. Health Sci. (2014) 1:19–23. Available online at: https://www.um.edu.mt/library/oar//handle/123456789/5072

[21] Cachia J. “*Breaking Silos, Building Bridges”: Annual Report 2017*. (2018). Available online at: https://www.parlament.mt/media/98685/dokument-17.pdf (accessed April 25, 2023).

[22] Cachia J. “*Empowering Stakeholders: Building Bridges and Crossing Them Together”: Annual Report 2018*. (2019). Available online at: https://healthservices.gov.mt/en/CommMentalHealth/Documents/Annual%20Report%202018.pdf (accessed March 3, 2023).

[23] Azzopardi-Muscat N Buttigieg S Calleja N. Merkur S. ‘*Malta: Health System Review', Health Syst*. (2017) 19:1–137. 28485715

[24] Buttigieg G. The mental health act 2012. J MaltColl Fam Dr. (2014)3:18–21.

[25] Camilleri M Sammut A Cachia J. The development of public mental health in Malta. Public Health Malt. (2019) 2:29–34.

[26] Cachia J. “*Mental Health and Wellbeing – Challenges and Opportunities”: Annual Report 2020*. (2022). Available online at: https://healthservices.gov.mt/en/CommMentalHealth/Documents/2022/Annual%20Report%202020%20Full.pdf (accessed March 4, 2023).

[27] Maltese Association of Psychiatry. 2018 Report on Staffing Levels in Malta's NHS, and Comparison with Recommended Standards. (2018). Available online at: https://map.org.mt/wp-content/uploads/2019/05/5258831524e6e3a2350c6c09149f183a4ab0dbe1.pdf (accessed March 10, 2023).

[28] Collins RL Wong EC Breslau J Burnam MA. Cefalu M, Roth E. Social marketing of mental health treatment: California's mental illness stigma reduction campaign. Am J Public Health. (2019) 109:S3. 10.2105/AJPH.2019.305129 31242016 PMC6595511

[29] Deane L. Service user participation: contemporary issues and obstacles for the national service users executive and service user participation. Crit Soc Think Policy Prac. (2011) 3:70–83.

[30] Heney D. Solving for stigma in mental health care. J Eval Clin Pract. (2022) 28:5. 10.1111/jep.13735 35809228

[31] Alliance for Mental Health. “*Mental Health Services in Malta: A Position Paper.”* (2016). Available online at: https://map.org.mt/2016/10/09/a4mh-position-paper-on-mental-health-services-in-malta/ (accessed March 13, 2023).

